# A Three-Metabolic-Genes Risk Score Model Predicts Overall Survival in Clear Cell Renal Cell Carcinoma Patients

**DOI:** 10.3389/fonc.2020.570281

**Published:** 2020-10-22

**Authors:** Yiqiao Zhao, Zijia Tao, Xiaonan Chen

**Affiliations:** Department of Urology, Shengjing Hospital of China Medical University, Shenyang, China

**Keywords:** clear cell renal cell carcinoma (ccRCC), risk score, TCGA, bioinformatics, metabolic gene

## Abstract

Metabolic alterations play crucial roles in carcinogenesis, tumor progression, and prognosis in clear cell renal cell carcinoma (ccRCC). A risk score (RS) model for ccRCC consisting of disease-associated metabolic genes remains unidentified. Here, we utilized gene set enrichment analysis to analyze expression data from normal and tumor groups from the cancer genome atlas. Out of 70 KEGG metabolic pathways, we found seven and two pathways to be significantly enriched in our normal and tumor groups, respectively. We identified 113 genes enriched in these nine pathways. We further filtered 47 prognostic-related metabolic genes and used Least absolute shrinkage and selection operator (LASSO) analysis to construct a three-metabolic-genes RS model composed of *ALDH3A2, B3GAT3*, and *CPT2*. We further tested the RS by mapping Kaplan-Meier plots and receiver operating characteristic curves, the results were promising. Additionally, multivariate Cox analysis revealed the RS to be an independent prognostic factor. Thereafter, we considered all the independent factors and constructed a nomogram model, which manifested in better prediction capability. We validated our results using a dataset from ArrayExpress and through qRT-PCR. In summary, our study provided a metabolic gene-based RS model that can be used as a prognostic predictor for patients with ccRCC.

## Introduction

Renal cell carcinoma (RCC) is a frequently diagnosed cancer and represents ~5 and 3% of all cancers in men and women, respectively (1, 2). The incidence of RCC has increased annually over the past 20 years (3). Among the histologic types of RCC, clear cell renal cell carcinoma (ccRCC) is the most common one (80–90%). At present, surgery is the gold standard for the treatment of localized ccRCC (4). Nonetheless, around one third of patients with ccRCC relapse (5). Over the past two decades, the development of ccRCC prognosis has only marginally improved (6). Investigation of the molecular mechanisms involved in ccRCC would benefit the development of new therapeutic strategies.

Dysregulated metabolism is considered as a hallmark for the progression and prognosis of various diseases (7–10). The diagnosis of RCC is usually suggested by systemic rather than urologic manifestations (11). As a result, RCC is often studied from a metabolic perspective. For example, ccRCC is reported to be related to the biallelic loss of the Von Hippel-Lindau tumor suppressor gene, which can lead to various metabolic alterations (12). In addition, mutations in RCC-associated genes are thought to be involved in pathways such as the tricarboxylic acid (TCA) cycle and tumor energetics (13). Contrastingly, Li et al. studied the relationships of metabolism-associated genes deregulation in ccRCC (14) and Luo et al. identified nine genes with risk scores associated with ccRCC prognosis (15). Some of these genes, such as CEP55, BIRC5, and CDC20 were found to be enriched in metabolic pathways. Nevertheless, relationship between ccRCC prognosis and an easy and practical risk score (RS) model composed of metabolism-related genes remains poorly understood.

The purpose of this study was to identify a metabolic-gene RS model based on The Cancer Genome Atlas (TCGA) by Gene Set Enrichment Analysis (GSEA) analysis, and to delineate its association with other clinicopathological characteristics and prognosis.

## Materials and Methods

### Data Preparation

**Figure 1:**
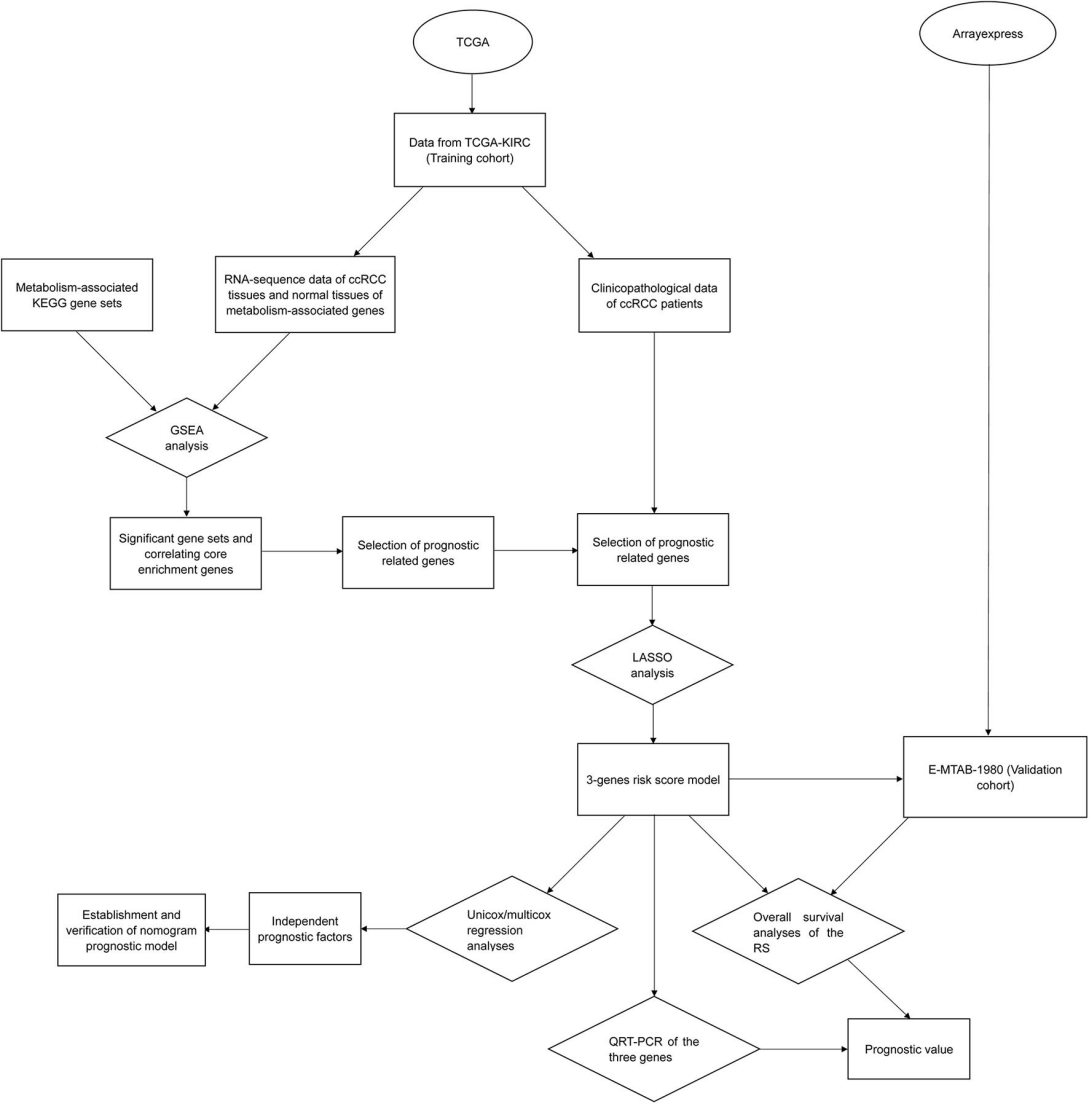
Overall workflow of the study design and analysis.

The overall workflow of the current was presented according to Figure 1. Gene expression information (FPKM, fragments per kilobase per million, including 72 normal samples and 539 tumor samples) and corresponding clinicopathological data of ccRCC patients were collected from TCGA (https://cancergenome.nih.gov/) as the training set (the clinical data of a total of 537 ccRCC patients were downloaded, however, patients with ambiguous clinical information such as “unknown” or “Tx,” “Mx” were not included in this study, besides, the clinicopathological characteristics “N stage” contains so many “Nx,” so it was also not studied in this research, finally, we incorporated 486 patients for the following investigations). Moreover, we downloaded 70 metabolism-associated gene sets from the gene sets database of Kyoto Encyclopedia of Genes and Genomes (KEGG) pathways in GSEA website (http://software.broadinstitute.org/gsea/downloads.jsp#msigdb). We also got ccRCC patients' (*N* = 101) RNA expression and clinical data of E-MTAB-1980 (*N* = 101) from ArrayExpress (https://www.ebi.ac.uk/arrayexpress/), which was applied as the validation cohort.

### Prognostic Metabolic Genes

A total of 1,465 genes were involved in 70 metabolism-related gene sets, among which the expression data of 1,413 genes were available in TCGA. We analyzed these data by GSEA software (version 4.0.3), and pathways that were significantly enriched in either tumor group or normal group were selected by false discovery rate (FDR) and nominal *p*-value, the number of permutations was set 1,000 times for each analysis. Genes contributing to the core enrichment were extracted for univariate COX analysis operated by “survival” package in R software (version 3.5.1), genes with *p* < 0.01 and hazard ratio (HR) >1 or <1 were sorted as prognostic core enrichment genes.

### Construction and Evaluation of the LASSO RS Model

Least absolute shrinkage and selection operator (LASSO) analysis of the prognostic metabolic genes with the help of “survival” and “glmnet” packages in R software, eventually, an RS was obtained in the form of:

RS = ∑n i=1 Coefi * xi

The xi represented the level of each component genes and Coefi were their corresponding coefficients, we defined high-risk and low-risk groups based on the median level of this RS in ccRCC patients. A Kaplan-Meier plot (K-M plot) of these two groups as along with 3- and 5-year survival receiver operating characteristic (ROC) curves were mapped and the area under ROC curves (AUC) value was utilized to assess the prognostic value and power of the RS.

To estimate the relationship of the RS and other clinicopathological characteristics, comparisons about levels of RS in different grades as well as T and M stages were engaged by scatter diagrams based on unpaired *t*-test which were constructed by GraphPad Prism 7, *p* < 0.05 was considered as significant. In addition, we also conducted 5-year survival ROC analyses of T/M stage and grade to evaluate their statistical power (The variable “stage” was not incorporated due to its ambiguity and its impact on mutual independence with other factors). On the other hand, univariate and multivariate Cox regression analyses were applied, and the *p*-value and HR were used to identify the independent prognostic factors.

### Nomogram

We built a nomogram consisting of independent prognostic factors for the prediction for the 3- and 5-year OS of ccRCC. ROC curves and calibration curves for 3- and 5-years overall survival (OS) were also mapped for verification of the power and accuracy of prognosis-prediction, furthermore, concordance-index (C-index) and its 95% confidence interval (CI) were calculated for justification of the nomogram model.

### Validation of the RS

A validation dataset, E-MTAB-1980 (*N* = 101) was downloaded from Arrayexpress (https://www.ebi.ac.uk/arrayexpress/). We realized that the data was log2 normalized, we reversely adjusted the data so that it was in line with that of TCGA. We applied the RS from the training set to validation set and divided the validation set into high- and low-risk groups by median RS. Eventually, a K-M plot and 3- and 5-year survival ROC curves were plotted.

For further validation, we compared the expression level of metabolic genes in the RS model in normal group and tumor group from TCGA. In addition, we performed quantitative real-time PCR (qRT-PCR) of the component genes in 16 pairs of ccRCC samples and matched adjacent normal kidney tissues (the expression data were integrated in Supplementary Tables 1A–C, respectively). Detailed procedures were reported in our previous research (16).

### Statistical Analysis

Univariate cox regression analyses were performed to identify the prognostic metabolic genes (*p* < 0.01) and prognostic clinicopathological factors (*p* < 0.05). Variables with significant values (*p* < 0.05) from univariate analyses were enrolled into the multivariate analyses for selection of independent prognostic factors. Kaplan-Meier plots were plotted through the application of “survival” package in R, log-rank test was used to analyze the survival data. *T*-tests were applied to visualize the distribution of the RS in patients grouped by vital variables like grade or T/M stage. For the evaluation of accuracy of the RS and the nomogram model, we did ROC analyses, and an AUC > 0.7 was respected to have acceptable predictive value. Differences were considered statistically significant at *p* < 0.05 in the survival analyses and *t*-tests.

## Results

### Acquiring Core Enrichment Genes

**Figure 2:**
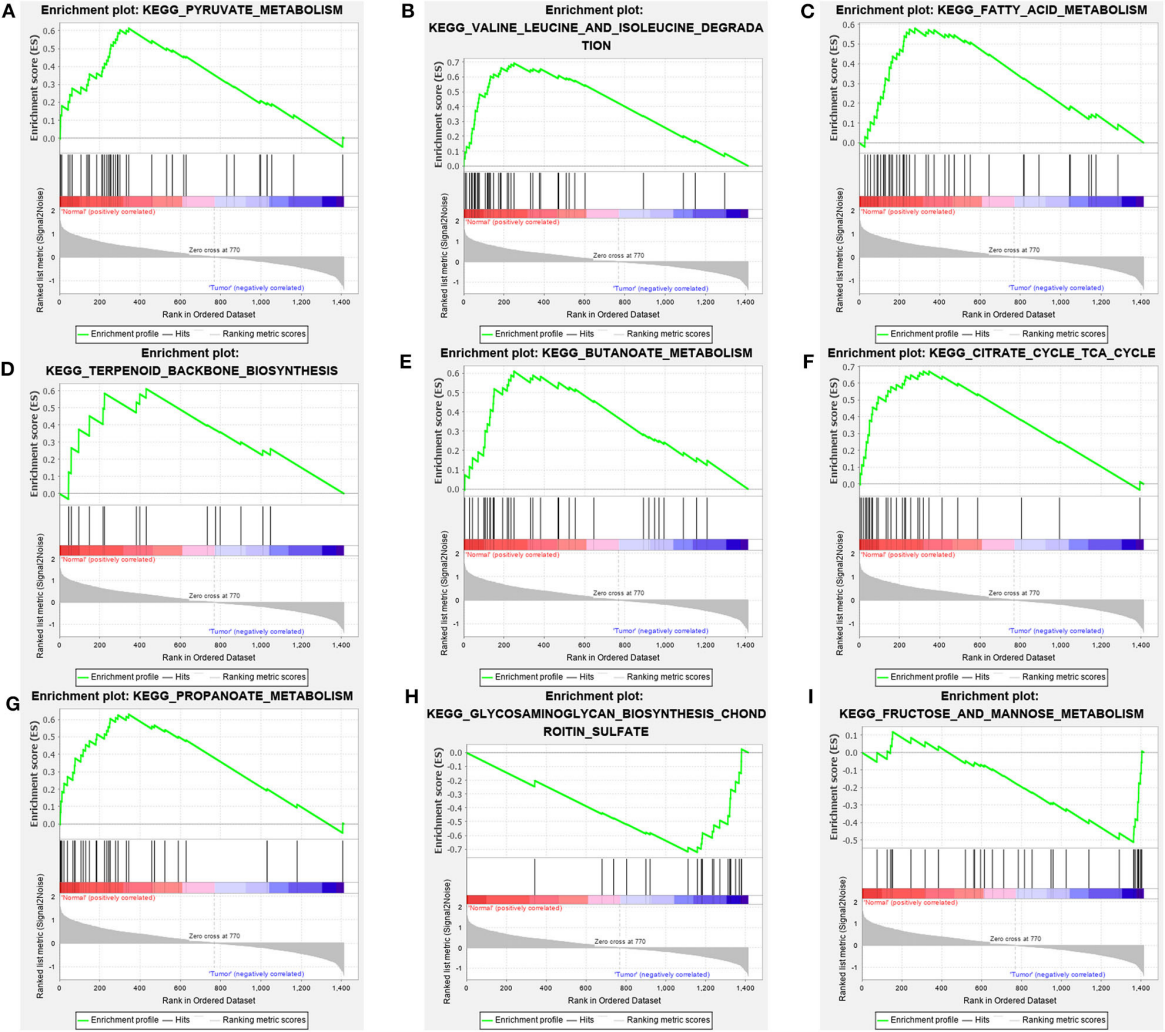
Metabolic pathways significantly enriched in normal groups **(A–G)** and ccRCC groups **(H,I)** based on GSEA of TCGA data.

GSEA analysis was applied via expression data of TCGA-KIRC and the 70 gene sets correlated with metabolism. We filtered the gene sets by FD<25% and nominal *p*-value <5%, eventually, seven gene sets significantly enriched in normal group and two gene sets significantly enriched in tumor group were obtained (Figure 2). The core enrichment genes of all these nine gene sets were extracted and integrated into a total of 113 genes (Supplementary Table 2).

### Selection of Prognostic Metabolism-Related Genes

Univariate Cox survival analysis was performed for the 113 core enrichment genes we got, *p* < 0.01 and HR < 1 or HR > 1 were used to screen prognostic genes, we obtained 49 genes that satisfied these filter conditions. However, the results of GSEA and those of survival analysis of two genes, *ACACA* and *MPI*, showed opposite trend. Therefore, the other 47 genes were considered as prognostic genes (Supplementary Table 3).

### Building and Verifying the RS Model

**Figure 3:**
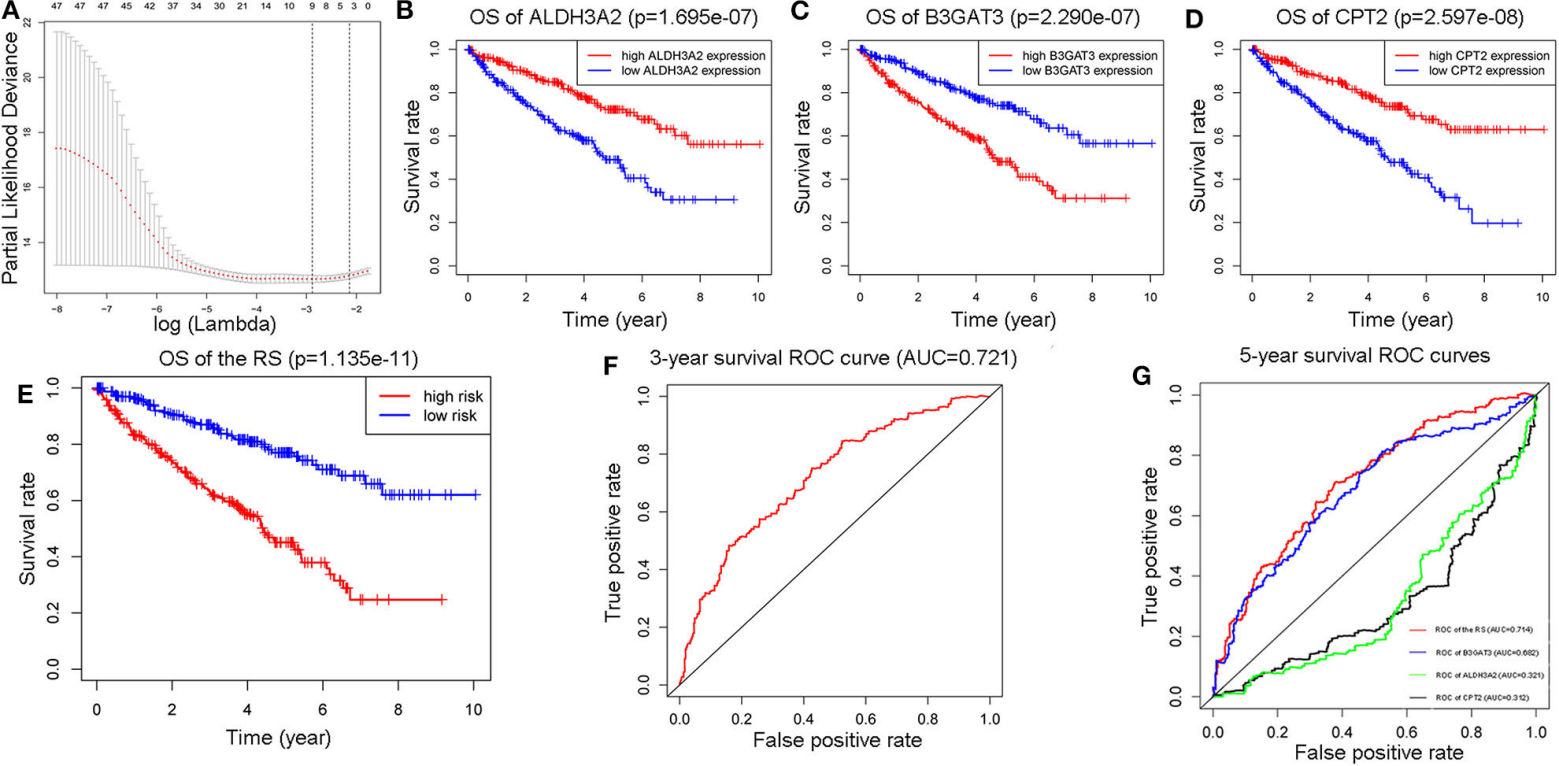
Construction of a three-metabolic-genes RS model from 47 prognostic metabolic genes **(A)**. Comparison of 5-year survival K-M plots of the three genes and the RS model they composed **(B–E)**, a 3-year ROC curve of the RS model **(F)**, and 5-year ROC curves of RS model along with those three genes **(G)**.

Through the application of “glmnet” and “survival” packages in R software, a three-gene-RS model was calculated (Figure 3A), which was presented as “(−0.00958307120509029) ^*^ (CPT2 expression) + (−0.00609212290247052) ^*^ (ALDH3A2 expression) + 0.0187929890446235 ^*^ (B3GAT3expression). Thereafter, 5-year OS Kaplan-Meier plots (K-M plots) of the RS and these three genes (Figures 3B–E) were mapped to verify the accuracy of the RS we got, as shown in the figure. The patients with ccRCC with lower levels of *ALDH3A2* and *CPT2* together with higher levels of *B3GAT3* had poorer prognosis, which was in line with our RS model. Additionally, the 3-and 5-year survival ROC curves of the RS (Figures 3F,G) displayed the relatively strong power of the RS (AUC = 0.721 and 0.714, respectively). Furthermore, we compared the power of the RS model with its three constituents, it was observed that the RS model had stronger predictability than its components, which showed that the significance of our RS over single genes.

### The Relationship Between RS and Other Clinicopathological Characteristics

**Figure 4:**
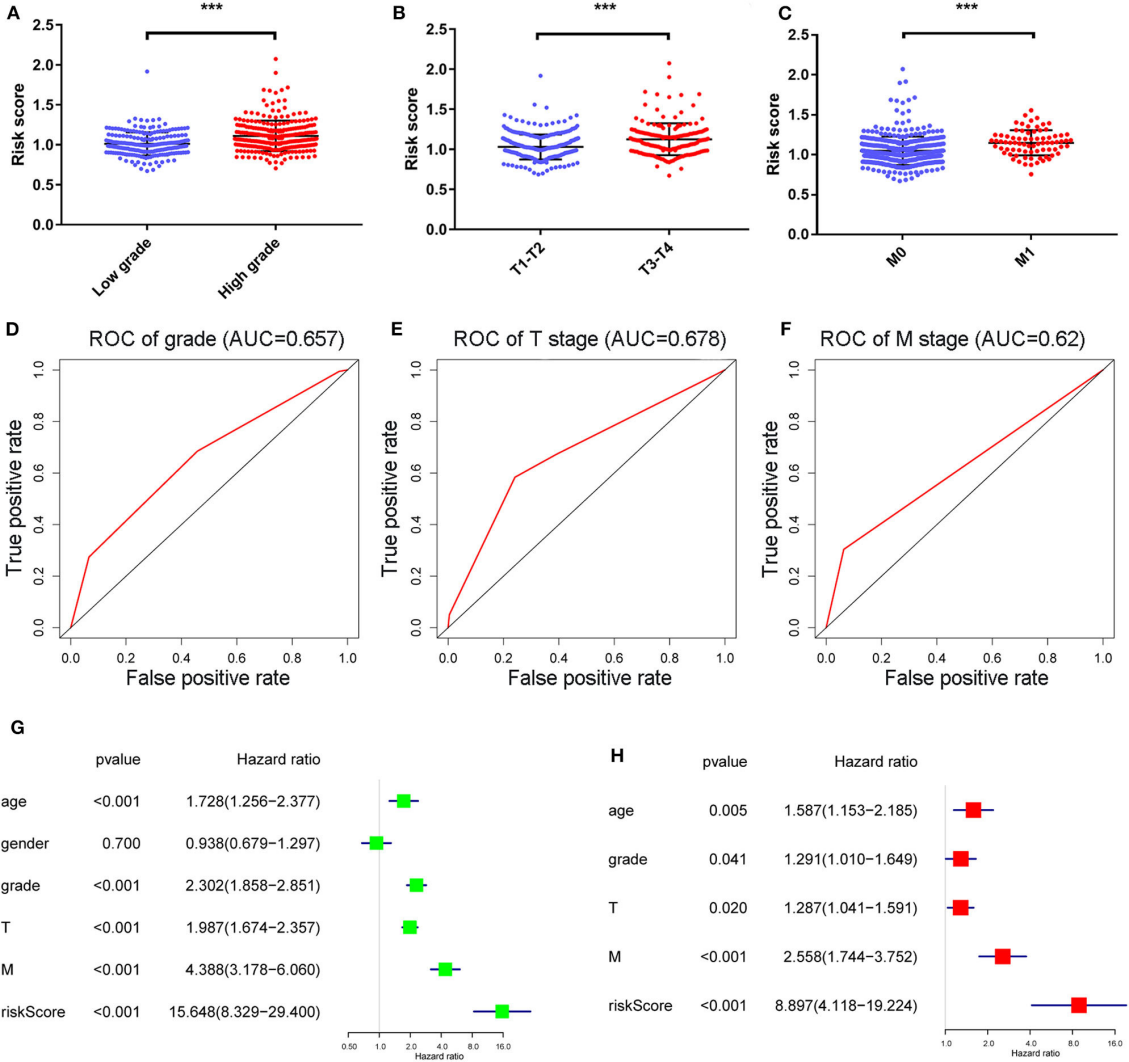
The distribution of RS in ccRCC patients with high and low grades, T stage and M stage **(A–C)** and their correlated 5-year survival ROC curves **(D–F)**. ***Means *p* < 0.01. Univariate and multivariate cox analyses of the RS model and other clinicopathological characteristics with overall survival **(G,H)**.

We further investigated the associations of the RS with other clinicopathological characteristics, and found that the distributions of the RS were remarkably different in low grade (G1-G2) and high grade (G3-G4), T stages and M stages (Figures 4A–C). We also noted that the corresponding 5-year survival ROC curves showed that the power of the RS w was better for prediction than these characteristics (Figures 4D–F, AUC = 0.657, 0.678, and 0.62, in respect).

**Figure 5:**
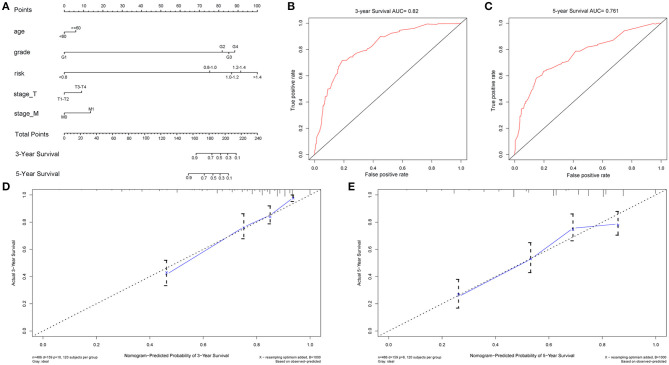
Nomogram model constructed by independent prognostic factors predicting 3- and 5-year OS for ccRCC patients **(A)**, their corresponding ROC curves **(B,C)**, and corresponding calibration curves **(D,E)** for validation of the model.

Univariate and multivariate Cox regression analyses (Figures 4G,H) revealed age, grade, T/M stages and the RS as independent prognostic factors (with *p*-value < 0.05 and HR > 1 in both analyses). Afterwards, a nomogram was constructed by these factors for better 3-and 5-year OS prediction (Figure 5A). The AUCs of corresponding ROC curves were 0.82 and 0.761, respectively (Figures 5B,C). The concordance-index (C-index) was 0.776 (95% [CI] 0.743–0.809). The calibration curves of the 3-and 5-year OS predicted by the nomogram model showed strong accordance with the outcomes that were observed (Figures 5D,E).

### Validation of the RS

**Figure 6:**
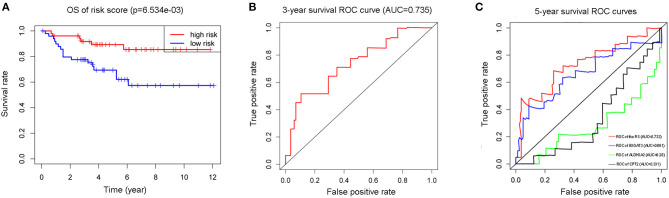
The K-M plot **(A)** and 3-year survival ROC curve **(B)** of the RS model, and the 5-year survival ROC curves of the RS model with its component genes: ALDH3A2, B3GAT3, and CPT2 **(C)** in validation cohort E-MTAB-1980.

**Figure 7:**
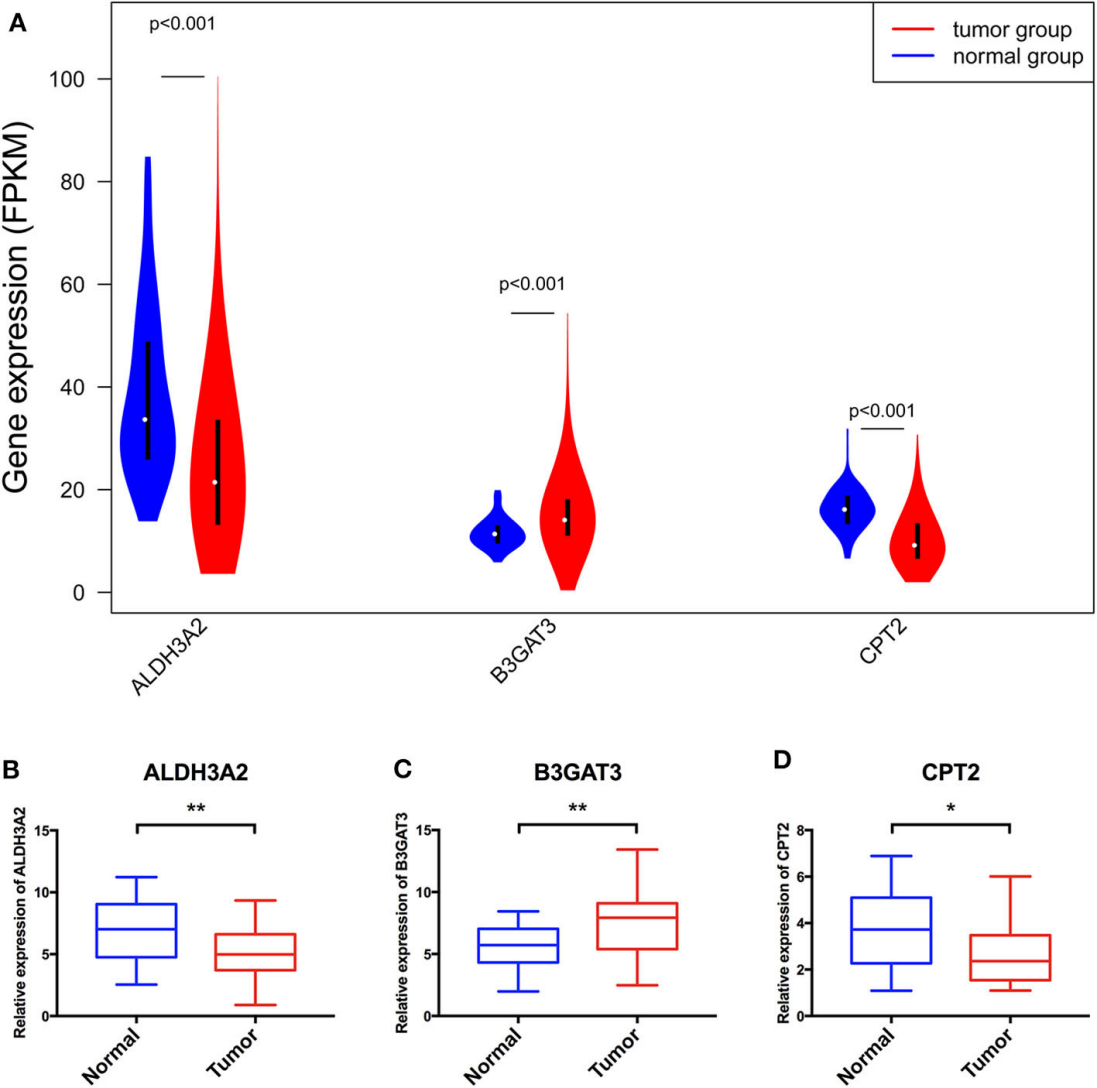
The violin plot based on TCGA data **(A)** and further validation via qRT-PCR **(B–D)** of the three genes consisting of the RS model. *Means *p* < 0.05, **Means *p* < 0.01.

In addition to the training cohort from TCGA, a validation cohort from the Arrayexpress, E-MTAB-1980 was utilized for validation (The clinical information of patients of these two cohorts were listed as Supplementary Tables 4, 5). The *p*-value of the OS was <0.05 and AUCs of the 3-and 5-year OS ROC curves were all >0.7 (Figures 6A–C). The violin plot (Figure 7A) based on TCGA data displayed remarkably different expression levels of ALDH3A2, B3GAT3, and CPT2 in tumor group compared with that of the normal group. The qRT-PCR results of these three metabolic genes (Figures 7B–D) gold standard for treatment were in line with those we got with the training cohort. The validation results indicated the reliability of the RS model.

### Contrast of the RS Model Obtained by Elastic Net Methods With That of LASSO

For the exploration of another RS model, we applied Elastic Net analysis and obtained a different RS model composed of six metabolic-genes to predict the 3- and 5-year OS of ccRCC patients from the training and validation cohorts. The results of TCGA patients (Supplementary Figures 1A,B) showed that the AUCs of 3- and 5-year OS were 0.725 and 0.738 respectively, besides, by data from the arrayexpress, the 3-year AUC was 0.738 and the 5-year AUC was 0.732 (Supplementary Figures 1C,D). The results were of similar significance as those of LASSO RS model, nevertheless, the LASSO model was constructed by 3 genes, which was fewer than the elastic model, which is more convenient for clinical use. Therefore, the LASSO model was chosen for further research.

## Discussion

CcRCC is a heterogeneous and lethal malignancy with poor prognosis (17). Despite its increasing incidence in the past two decades, the mortality of ccRCC patients has not decreased (3, 18, 19). Metabolic alterations are considered a central feature of ccRCC (20) with the reporting of potential interactions of ccRCC with various metabolic reprogramming components (21, 22). Additionally, several studies have examined the role of metabolic genes in the RCC processes. For example, Lucarelli et al. delineated the impact of *NDUFA4L2* on ccRCC bioenergetics and different biological processes (21), and Xiao et al. identified that *HAO2* promotes lipid catabolic and metabolic processes and lipid oxidation in ccRCC (23). However, a metabolic gene signature for the prediction of patients' prognosis has been established.

In the current study, we used GSEA analysis to analyze the expression data of 1,413 metabolic genes obtained from 70 KEGG metabolic pathways from 72 normal and 539 ccRCC samples. These samples were acquired from TCGA dataset. Nine out of 70 metabolic pathways identified were relevant to differentiation status of the patients with ccRCC, and 113 genes were significantly enriched in these nine pathways. These were integrated as core enrichment genes (we selected 47 metabolic genes) and operated on using LASSO analysis. Thereafter, a three metabolic-genes RS model was generated, which could predict the OS of patients with ccRCC. The multivariate Cox regression analyses delineated age, grade, T/M stages and the RS as fiveindependent prognostic factors. A nomogram model, built using these three factors showed high accuracy in its prediction. We then validated the RS model by Arrayexpress dataset E-MTAB-1980 and by qRT-PCR. Our results validated the predicting ability of the RS model.

The RS model was made up of *ALDH3A2, B3GAT3*, and *CPT2*. As far as we know, the relationship among these three genes with ccRCC has not been studied previously. ALDH3A2 is predominantly localized to the liver and is expressed in the kidney (24). It is downregulated in colon cancer cells and involved in its glycolysis and gluconeogenesis pathways (25). Our study showed that low levels of ALDH3A2 were associated with decreased survival. This may be through ALDH3A2's involvement in the pyruvate or propanoate metabolism. The induction of pyruvate dehydrogenase kinase may cause pyruvate mitochondrial suppression in ccRCC cells and the interference of pyruvate metabolism may be a potential therapeutic strategy (26). Alternatively, pyruvate and propanoate metabolism were both identified to be downregulated in ccRCC cells (27). B3GAT3 was found to participate in proteoglycan biosynthesis (28). B3GAT3 is highly expressed in liver cancer cells and considered a potential prognostic biomarker (29). Here, we demonstrated that the higher expression of B3GAT3 could cause poorer OS in patients with ccRCC. As it mainly functions in glycosaminoglycan biosynthesis of chondroitin sulfate in ccRCC cells, several genes associated with this metabolism were upregulated in ccRCC cells. A score has also been constructed based on the glycosaminoglycan profiles as a diagnostic biomarker (30). CPT2 is involved in SCD1-mediated lipid metabolism and acylcarnitine-mediated STAT3 activation in liver carcinogenesis (28, 31). In addition, CPT2 is thought of as a mitochondrially-expressed protein that is involved in the fatty acid degradation pathway functioning in the epithelial ovarian carcinoma (32). In this study, CPT2 was also related to fatty acid metabolism and its higher expression might lead to worse OS in patients with ccRCC. Fatty acid metabolism plays integral roles in the ccRCC tumorigenesis (33) and the prognosis of metastatic RCC in patients (34). These are in accordance with our results and highlight the reliability of our study.

There are a lot of grouped variable selection methods, such as LASSO method Ridge methods and Elastic Net method. Among these three methods, The RS model by Ridge method was composed of all 47 metabolic genes, which were too much for application. Intriguingly, we performed Net method using all clinical variables in ccRCC as well as Elastic Net analysis on a web-based tool “ESurv” [https://easysurv.net; (35)]. The prediction performances (Supplementary Figures 2A,B, respectively) of prediction signatures of these two methods were much better than our results, however, the gene signatures predicted by this web-tool consists of 39 genes for Net (Supplementary Table 6) and 159 genes for Elastic Net (Supplementary Table 7), the amount of genes were too much so they were challenging for clinical use. Besides, the genes involved in these signatures were not restricted to metabolic genes, thus, at last, we determined LASSO model as final choice. Although ESurv was not suitable for our current study, we see enormous possibility of the prognostic signatures we calculated by this tool, thence, we would like to apply it in our future study when studying other prognostic gene sets.

Despite our promising results, there are several limitations that should be considered as outlined below. First, increasing the number of cohorts with sample capacity can aid the validation of the three-metabolic-genes RS model. The number of normal and tumor samples was 72 and 539, respectively. This imbalance in sample numbers may affect the reliability of our results. In addition, this study mainly relied on *in silico* analyses and further validation using *in vitro* and *in vivo* experiments as well as clinical data are warranted. Besides most of the patients involved in this study were Americans as observed from the available data through the curated databases. As a consequence, the RS model may not pertain to patients with ccRCC from other countries and ethnicities. On the other hand, the cutoff value is different in each cohort because they used median value as a cutoff.

In summary, we identified some metabolic pathways that may function in ccRCC cells and built a clinically practical and easily implemented RS model composed of three metabolic genes. This model can become a potential biomarker for predicting the prognosis of patients with ccRCC. This model can also enable the delineation of the molecular mechanisms involved in ccRCC.

## Data Availability Statement

The datasets presented in this study can be found in online repositories or in supplementary materials. The names of the repository and accession number/the data generated by human participants can be found in the article/Supplementary Material.

## Ethics Statement

The studies involving human participants were reviewed and approved by Ethics Committee of Shengjing Hospital. The patients/participants provided their written informed consent to participate in this study.

## Author Contributions

XC and YZ conceived and designed the study. YZ contributed to the mining and analyses of the data. XC and ZT drafted and revised the manuscript. ZT and YZ prepared figures and/or tables. XC edited the manuscript. All authors contributed to the article and approved the submitted version.

## Conflict of Interest

The authors declare that the research was conducted in the absence of any commercial or financial relationships that could be construed as a potential conflict of interest.
