# Supplementary material for: A Three-Metabolic-Genes Risk Score Model Predicts Overall Survival in Clear Cell Renal Cell Carcinoma Patients
Source: Front Oncol. 2020 Oct 22;10:570281. doi: 10.3389/fonc.2020.570281 (PMC7642863; doi:10.3389/fonc.2020.570281)
Supplement: Supplementary file 4 [file Table_4.docx]

Table S4: General characteristics of the patients involved in training cohort

| Characteristics | N（%） |
| --- | --- |
| Age （years） |  |
| <60 | 226 (46.5) |
| ≥60 | 260 (53.5) |
| Gender |  |
| Male | 322 (66.3) |
| Female | 164 (33.7) |
| T stage |  |
| T1 | 242 (49.8) |
| T2 | 61 (12.6) |
| T3 | 172 (35.4) |
| T4 | 11 (2.2) |
| M stage |  |
| M0 | 409 (84.2) |
| M1 | 77 (15.8) |
| Histological grade |  |
| G1 | 10(2.1) |
| G2 | 210 (43.2) |
| G3 | 194 (39.9) |
| G4 | 72 (14.8) |
