# Supplementary material for: A Three-Metabolic-Genes Risk Score Model Predicts Overall Survival in Clear Cell Renal Cell Carcinoma Patients
Source: Front Oncol. 2020 Oct 22;10:570281. doi: 10.3389/fonc.2020.570281 (PMC7642863; doi:10.3389/fonc.2020.570281)
Supplement: Supplementary file 5 [file Table_5.docx]

Table S5: General characteristics of the patients involved in validation cohort

| Characteristics | N（%） |
| --- | --- |
| Age （years） |  |
| <60 | 41 (40.6) |
| ≥60 | 60 (59.4) |
| Gender |  |
| Male | 77 (76.2) |
| Female | 24 (23.8) |
| T stage |  |
| T1 | 68 (67.3) |
| T2 | 11 (10.9) |
| T3 | 21 (20.8) |
| T4 | 1 (1) |
| M stage |  |
| M0 | 89 (88.1) |
| M1 | 12 (11.9) |
| Fuhrman grade |  |
| G1 | 13(12.8) |
| G2 | 59 (58.4) |
| G3 | 22 (21.8) |
| G4 | 5 (5) |
| Undetermined | 2 (2) |
