# Supplementary material for: A Three-Metabolic-Genes Risk Score Model Predicts Overall Survival in Clear Cell Renal Cell Carcinoma Patients
Source: Front Oncol. 2020 Oct 22;10:570281. doi: 10.3389/fonc.2020.570281 (PMC7642863; doi:10.3389/fonc.2020.570281)
Supplement: Supplementary file 8 [file Table_8.docx]

Table S1: Relative expression level of ALDH3A2, B3GAT3 and CPT2 in 16 pairs of ccRCC samples and matched adjacent normal kidney tissues.

Table S2: 113 core enrichment genes from significant KEGG metabolic pathways (FDR<25% and nominal p value<5%)

Table S3: Selection of 47prognostic metabolic genes by certain cut-off standard (p<0.01 and HR<1 or >1)

Table S4: General characteristics of the patients involved in training cohort.

Table S5: General characteristics of the patients involved in validation cohort.

Table S6: Estimated coefficients of signatures predicted by “Net” method.

Table S7: Estimated coefficients of signatures predicted by “ENet” method.

Figure S1: Time-dependent ROC curves of RS model constructed by elastic net analysis, (A) and (B) depicted the AUCs of 3-year and 5-year OS of ccRCC patients in training cohort, on the other hand, (C) and (D) represent the AUCs of 3- and 5-year OS of ccRCC patients in the validation cohort.

Figure S2: AUC values of ROC curves obtained via web-based tool ESurv, the X-axis means the follow-up time and the Y-axis represents the corresponding AUCs. According to the results, the AUCs of variable signatures calculated by “Net” and “ENet” methods (A and B, in respect) were remarkably high (AUC>0.8).
