# Supplementary figures and images for: A Three-Metabolic-Genes Risk Score Model Predicts Overall Survival in Clear Cell Renal Cell Carcinoma Patients
Source: Front Oncol. 2020 Oct 22;10:570281. doi: 10.3389/fonc.2020.570281 (PMC7642863; doi:10.3389/fonc.2020.570281)

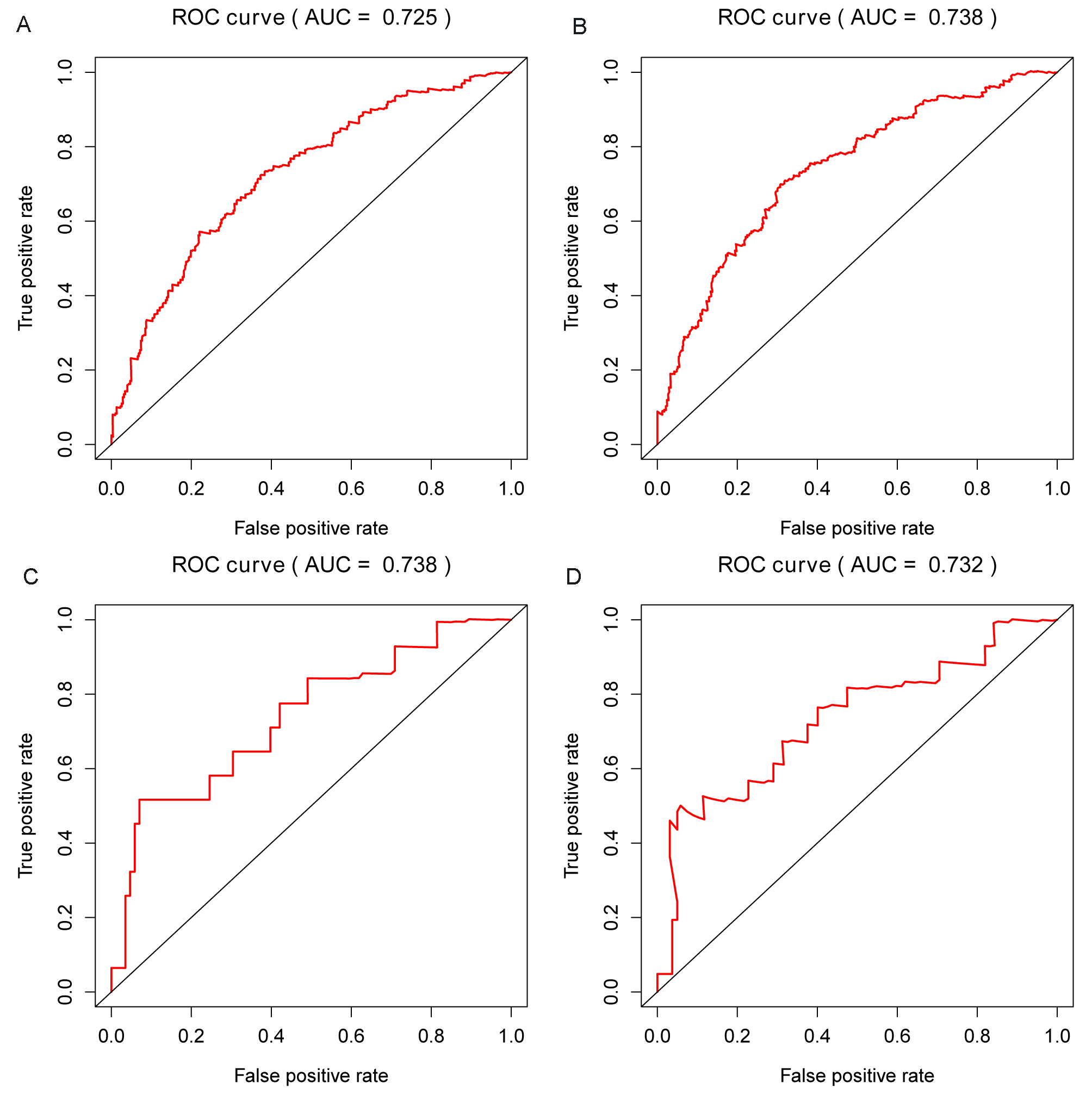

Supplement: Supplementary file 9 [file Image_1.tif]

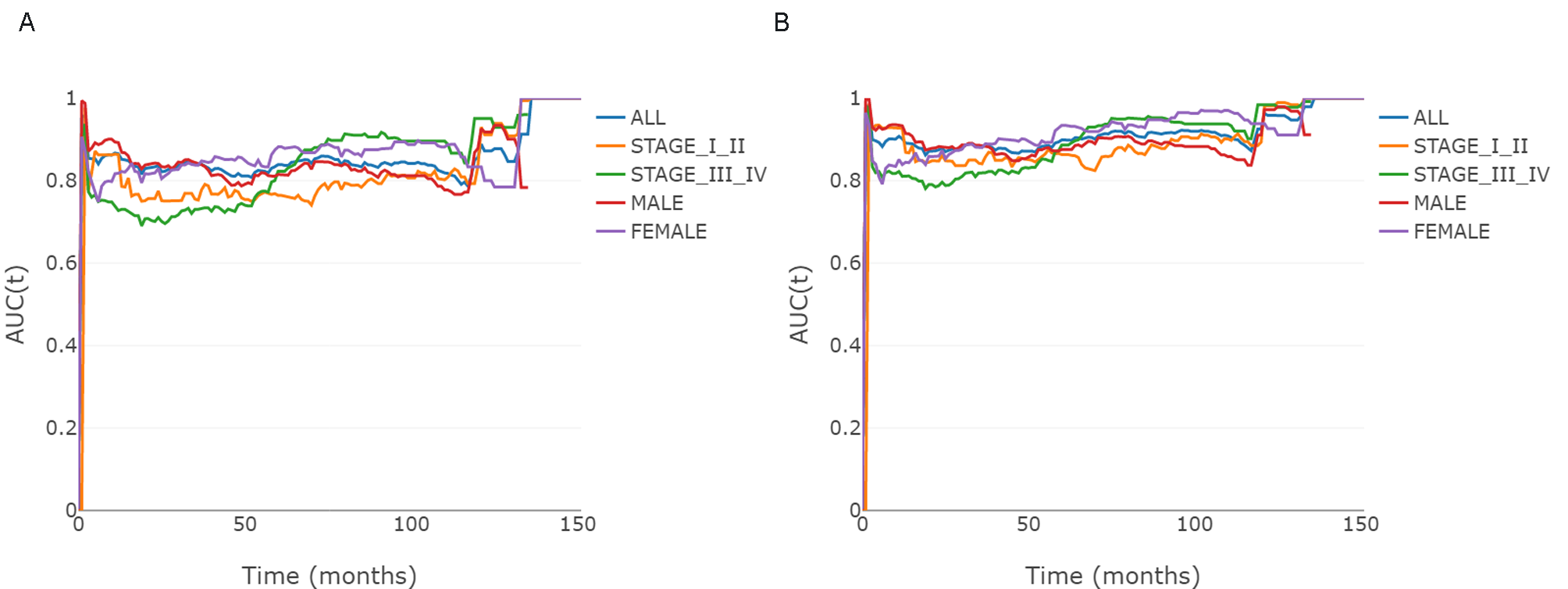

Supplement: Supplementary file 10 [file Image_2.tif]
